# TREK2 Lipid Binding Preferences Revealed by Native Mass Spectrometry

**DOI:** 10.1021/jasms.4c00112

**Published:** 2024-06-06

**Authors:** Lauren Stover, Yun Zhu, Samantha Schrecke, Arthur Laganowsky

**Affiliations:** † Department of Chemistry, Texas A&M University, College Station, Texas 77843, United States

**Keywords:** native mass spectrometry, protein−lipid interactions, membrane proteins, ion channel

## Abstract

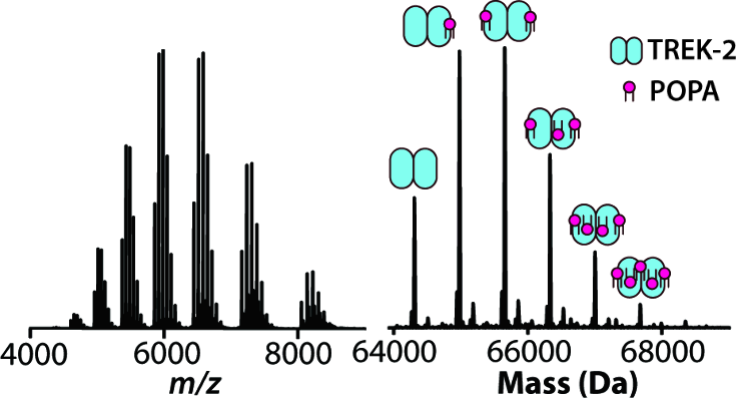

TREK2, a two-pore domain potassium channel, is recognized for its regulation by various stimuli, including lipids. While previous members of the TREK subfamily, TREK1 and TRAAK, have been investigated to elucidate their lipid affinity and selectivity, TREK2 has not been similarly studied in this regard. Our findings indicate that while TRAAK and TREK2 exhibit similarities in terms of electrostatics and share an overall structural resemblance, there are notable distinctions in their interaction with lipids. Specifically, SAPI(4,5)P2,1-stearoyl-2-arachidonoyl-*sn*-glycero-3-phospho-(1′-myo-inositol-4′,5′-bisphosphate) exhibits a strong affinity for TREK2, surpassing that of dOPI(4,5)P2,1,2-dioleoyl-*sn*-glycero-3-phospho-(1′-myo-inositol-4′,5′-bisphosphate), which differs in its acyl chains. TREK2 displays lipid binding preferences not only for the headgroup of lipids but also toward the acyl chains. Functional studies draw a correlation for lipid binding affinity and activity of the channel. These findings provide important insight into elucidating the molecular prerequisites for specific lipid binding to TREK2 important for function.

## Introduction

Two-pore domain potassium channels (K_2P_) are a family of leak channels that regulate cell neuronal excitability and maintain the resting membrane potential.^1^ The three members of the TREK (TWIK RElated K+ channel) subfamily, TREK1, TREK2, and TRAAK, are expressed abundantly in both the central and peripheral nervous systems. TREK channels have important roles in physiological and pathological processes where their function has been associated with depression, pain perception, anesthesia, and neuroprotection.^2,3^ Previous studies have shown TREK2 is responsible for maintaining the background potassium current in dorsal root ganglia. More specifically, TREK2 hyperpolarizes the membrane potential of the c-fiber nociceptors, limiting pain sensation by controlling the spontaneous firing rate. Knockdown of TREK2 results in an increase in measured pain in rats.^2,4^ The opening of TREK2 has also been shown to protect cells against apoptosis associated with high intrarenal pressure.^5^

TREK2 channels are regulated by many factors including temperature, pH, membrane stretch, and voltage.^6^ These channels can even detect a pressure profile asymmetry within the lipid bilayer, responding differently to changes in both the inner and outer leaflet. This allows the channel to be dynamic and able to respond to real physiological changes in the membrane.^7^ TREK2 is responsive to not only the membrane stretch but also the composition of the membrane, such as activation by long-chain polyunsaturated free fatty acids (PUFAs). TREK2 has been shown to discriminate the double bond isomers of the unsaturated fatty acids with structural specificity.^8^ A recent study has shown that TRAAK selectively binds lipids and can discriminate the fatty acid linkage in the *sn*-1 position.^9^

Lysophospholipids with a bulky polar headgroup and long acyl chains can activate TREK and TRAAK channels.^10^ Bulky headgroups, such as lysophosphatidylcholine (LPC) and lysophosphatidylinositol (LPI), had a more pronounced effect on channel opening than arachidonic acid. However, activation by LPC relies on the integrity of the cell, suggesting lysolipids may not activate TREK and TRAAK through direct interaction.^10^ Furthermore, whether the lysolipid is located on the inner or the outer leaflet of the bilayer also makes a difference. LPC activates TREK from the extracellular side while lysophosphatidic acid (LPA) activates from the intracellular layer.^10,11^ LPC even becomes inhibitory in the inner leaflet.^10^ Phospholipids, such as phosphatidic acid (PA) and phosphatidylserine (PS), can also alter TREK channel activity.^12^ Phosphatidylinositol-4,5-bisphosphate (PI(4,5)P_2_) can either activate or inhibit TREK1 depending on the intracellular pH and location in the bilayer.^12−15^ Insertion of PIP_2_ into the intracellular side of the membrane restores TREK1 activity.^12,15^ The interaction between the C-terminus of TREK1 (and part of TM4) and PI(4,5)P_2_ is thought to be nonspecific electrostatic binding.^16^

TREK2 contains three predicted N-linked glycosylation sties. Glycosylation is an important post-translational modification that has functional roles in the maturation of membrane proteins,^17^ such as regulating conformational dynamics and interactions.^18^ Although N-linked glycans share a common trimannosyl-chitobiose core, (mannose)_3_(*N*-acetyl glucosamine)_2_ (Man_3_GlcNAc_2_), the type, degree of core fucosylation, and branching are strongly influenced by protein structure, which determines the accessibility of glycosylation site(s).^19^ Glycan heterogeneity stems from the decoration of the glycan core with a small number of monosaccharide molecules, such as fucose and mannose, resulting in different glycoforms with an estimated ∼7000 structures.^20^

Native MS is an innovative biophysical technique for studying membrane proteins that preserves the noncovalent interactions between protein and ligands allowing binding constants and other biophysical parameters to be measured.^21^ One advantage of high-resolution MS over all other established methods to study membrane proteins is that mass differences can be resolved,^22,23^ such as copper ion binding to TRAAK.^24^ Post-translational modifications and the homogeneity of samples can also be easily assessed using native MS. In previous studies, native MS has revealed the selectivity between protein and lipids, the allosteric effect between two lipids, the stabilization of protein by lipids, and thermodynamic properties of proteins.^21,22,25−27^ Furthermore, native MS can also provide invaluable insight into guide structural studies, which has led to the determination of membrane proteins structures with resolved density for lipids.^27,28^

In this work, we use a combination of native MS and functional assays to determine the lipid binding preferences of TREK2 and how these interactions impact function. We optimized the expression of purification of TREK2, a glycosylated membrane protein, for native MS studies. The optimized samples enabled the ability to resolve small adducts, such as K^+^ binding. After screening a subset of lipids, we have found that the dissociation constant (*K*_D_) values indicate the channel is selective toward specific lipids.

## Experimental Section

### TREK2 Expression and Purification

TREK2 was expressed and purified from insect cells as previously described.^24^ In brief, TREK2 (Uniprot P57789-1, residues 55–335 with T58A mutation) was expressed with a C-terminal StrepTag-II (AddGene 191475). Mutations introduced to abolish specific N-linked glycan sites were introduced using the KLD reaction enzyme mix (NEB). Cell pellets containing overexpressed TREK2 were resuspended in KCl lysis buffer (50 mM TRIS, pH 7.4 RT, and 150 mM KCl) and passed through a microfluidizer 2–3 times. Insoluble material was pelleted by centrifugation at 20 000×*g* for 25 min at 4 °C prior to pelleting membranes by centrifugation at 100 000×*g* for 2 h at 4 °C. The membranes were resuspended and homogenized in KCl lysis buffer. TREK2 was extracted by the addition of 1% OGNG for 2 h. The sample was centrifuged for 10 min at max speed in a benchtop centrifuge, and the supernatant was loaded onto a StrepTrap (Strep-Tactin Sepharose, IBA Lifesciences) pre-equilibrated in loading buffer (KCl lysis buffer with 0.12% OGNG). The bound protein was washed with 5 CV of loading buffer and then eluted with the elution buffer (loading buffer containing 2.5 mM desthiobiotin). The eluted sample was loaded onto a HiTrap Desalting 26/10 column equilibrated with the loading buffer. EndoH was then added to the protein and incubated overnight at 4 °C. The sample was checked with mass spectrometry to ensure the glycans were removed, and if not, more EndoH was added. The sample was then loaded back onto a StrepTrap to remove EndoH. The protein was eluted and injected onto a Supderex 200 gel filtration column (Cytiva) equilibrated in GF buffer (50 mM TRIS, pH 7.4 at room temperature, 200 mM KCl, and 0.062% C10E5). The fractions corresponding to TREK2 were pooled, glycerol was added to a final concentration of 20%, and the protein was flash frozen in liquid nitrogen.

### Preparation of Lipids

Lipids were prepared as previously described.^26^ In short, lipids (Avanti) dissolved in chloroform were aliquoted and dried down to a film under a stream of nitrogen gas. Lipid films were resuspended in deionized water to a concentration around 3 mM. The concentrations of lipids were determined using a phosphorus assay.^29^ Lipid stocks were stored at −20 °C, and defrosted lipids were sonicated prior to use. The lipids were serially diluted to concentrations twice those desired to keep the protein concentration fixed at 1 μM across all titrations.

### Native Mass Spectrometry

Following previous methods,^30^ the purified protein was buffer exchanged into an aqueous ammonium acetate solution (200 mM ammonium acetate, 0.062% C10E5) using a centrifugal desalting column (Bio-Spin *P*6̅ Gel Columns, Bio-Rad) for native mass spectrometry studies. TREK2 samples were infused using static, nanoelectrospray ionization into the front end of the Thermo Scientific Exactive Plus Orbitrap with Extended Mass Range (EMR) and measurements performed at room temperature. The instrument was tuned as follows: source DC offset of 40, injection flatapole DC to 8.0 V, inter flatapole lens to 4, bent flatapole DC to 3, transfer multipole DC to 3, and C trap entrance lens to 0. The spray voltage was set to 1.5 kV, capillary temperature to 300 °C, and trapping gas pressure to 5.0 with the in-source CID to 50 and CE to 30. Mass spectra were acquired with settings of 35 000 resolution, microscans of 1, and averaging of 100.

### Liposome Flux Assay

The liposome flux assays were performed as previously described.^9^ In brief, molar ratios of the desired lipids were used and then dried down to a film under a stream of liquid nitrogen. This film was then washed with 2 volumes of pentane and dried under a stream of liquid nitrogen each time. The vials were places in a desiccator overnight. The next day, the lipid film was rehydrated in rehydration buffer (150 mM KCl, 20 mM HEPES, pH 7.4 RT) and sonicated in a water bath for 10 min. One hundred fifty microliters of the sonicated lipids was mixed with an equal volume of the rehydration mixture and then extruded using a miniextruder from Avanti. The 50 nM membrane filters (Cytiva) were used. The sample was extruded 51 times, and then the extruded liposomes were put into a fresh vial. One hundred fifty microliters was taken out of this vial and placed into a second vial. An equal volume of solubilization buffer (rehydration buffer with 20 mM DM) was added to these vials, and the vials were then allowed to rotate at room temperature until the mixture looked clear, at least 30 min. Once the solutions were clean, TREK2^N84Q^ treated with Kifunencin and EndoH (the same sample used in native MS studies) was added to the vial into which 150 μL of the sonicated lipids was added at a ratio of 1:100 w/w protein to lipid and allowed to rotate for at least 1 h at room temperature. In the meantime, BioBeads (Bio-Rad) were prepared by consecutive washes of different solvents. The solvent was added to the BioBeads, allowed to sit for 5 min, and then spun down, and the solvent was discarded. The washes were 1 column volume of methanol, followed by 5 column volumes of water, and finally 3 column volumes of rehydration buffer. BioBeads were then added to the vials and allowed to rotate until the solution was clear again, a minimum of 2 h at room temperature or overnight at 4 °C. Once the samples became cloudy again, the samples were extruded in the same manner as previously described. For the flux assay, a CLARIOstar (BMG LabTech) was used and the experiment performed at room temperature. Four wells were prepared at a time with 190 μL of the flux assay buffer (150 mM NaCl, 20 mM TRIS, pH 7.4 RT), 5 μL of ACMA, and the liposomes, both the control and the one containing protein, at 1% and 2% final concentration. These were added quickly and data collection started, collecting every 5 s for 1 min. Once finished, 10 μL of CCCP was added to each well and mixed, and data were collected every 5 s over the course of 7 min. Finally, 1 μL of valinomycin was added to each well, and data were collected every 5 s for 5 min.

## Results

### Optimization of TREK2 for Native MS

As TREK2 contains N-linked glycosylation sites, the first objective of our study was to optimize the expression construct and purification of the channel. Each subunit has three predicted N-linked glycan sites that are located in an extracellular, disordered loop. Given the locations, it is not anticipated that these PTMs will influence lipid binding. For these studies we selected a truncated form of TREK2 that has been used for structural and functional studies.^31^ We first prepared TREK2 in pentaethylene glycol monodecyl ether (C10E5), a detergent with ideal properties for native MS studies, such as releasing easily from membrane proteins and also reducing charge on protein complexes.^32^ The native mass spectrum of the sample shows the protein is decorated with heterogeneous glycans, which precludes us from resolving individual lipid binding events. To overcome this barrier, glycans are often removed enzymatically, or sites within the protein mutated to abrogate glycosylation, to obtain more homogeneous protein samples. TREK2 incubated with peptide-*N*-glycosidase (PNGase F), an enzyme that cleaves between the Asn residue and inner core GlcNAc,^33^ did not improve sample homogeneity.

**Figure 1:**
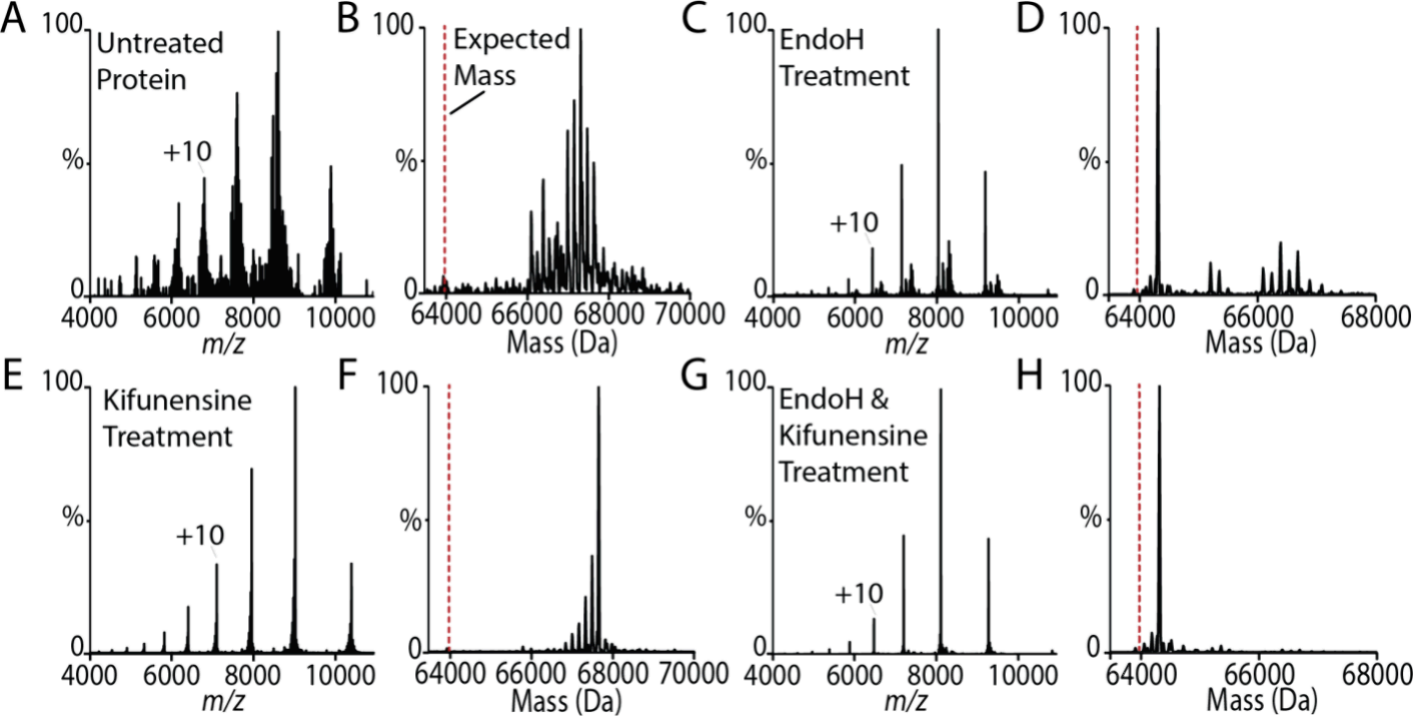
Optimization of TREK2^N84Q^ for native mass spectrometry. (A, B) Mass spectrum of TREK2^N84Q^ in C10E5. (B) Deconvolution of the mass spectrum is shown. (C, D) Mass spectrum of TREK2^N84Q^ after incubation with EndoH. (E, F) Mass spectrum of TREK2^N84Q^ expressed in the presence of kifunensine and after incubation with EndoH.

As PNGase F treatment was not efficient, we conducted mutagenesis studies focused on the three predicted N-linked glycosylation sites (N144, N147, and N148) of TREK2 to reduce the number of glycan sites. We expressed and purified TREK2 containing single (N144Q and N148Q) and double (N147Q, N148Q) mutations. Of the single substitutions, the N144Q mutant protein slightly improved sample heterogeneity while not impacting protein expression (Figure 1A). The native mass spectrum shows the different glycans vary in mass by ∼160 Da, corresponding to hexose. The sample was then treated with Endoglycosidase H (Endo H), which cleaves between the two innermost GlcNAc residues leaving a GlcNAc (203 Da) on the Asn side chain. While the main peak in the deconvoluted mass spectrum corresponds to TREK2^N84Q^ with and without the inner core GlcNac, there are other higher molecular species present, ranging from 1100 to 3590 Da in additional mass (Figure 1B). For simplicity, we will refer to TREK2^N84Q^ as TREK2.

We next explored the use of kifunensine, a potent mannosidase I inhibitor that leads to high mannose N-glycans that are sensitive to Endo H.^33−36^ This approach has successfully been used to produce deglycosylated material for structural studies. The native mass spectrum of TREK2 expressed in the presence of kifunensine (Figure 1C) has a series of peaks, each with a mass shift of 160 Da, corresponding to different numbers of mannose on the glycan, as expected for kifunensine treatment.^36^ The sample incubated with EndoH (Figure 1D) resulted in a sample that was largely homogeneous. Despite the presence of low abundant species that differ in mass, the main peak corresponds to a mass of 64 310 Da. The theoretical mass for the homodimer with no modifications is 63 794 Da. The additional mass corresponds to the remaining inner core GlcNAc, retained after EndoH cleavage, and potential modifications of the glycan, such as phosphorylation, sulfonation, and acetylation.^37^ Nevertheless, the optimized sample of TREK2 is most suitable for mass spectrometry studies.

### Characterization of Lipid–TREK2 Interactions

**Figure 2:**
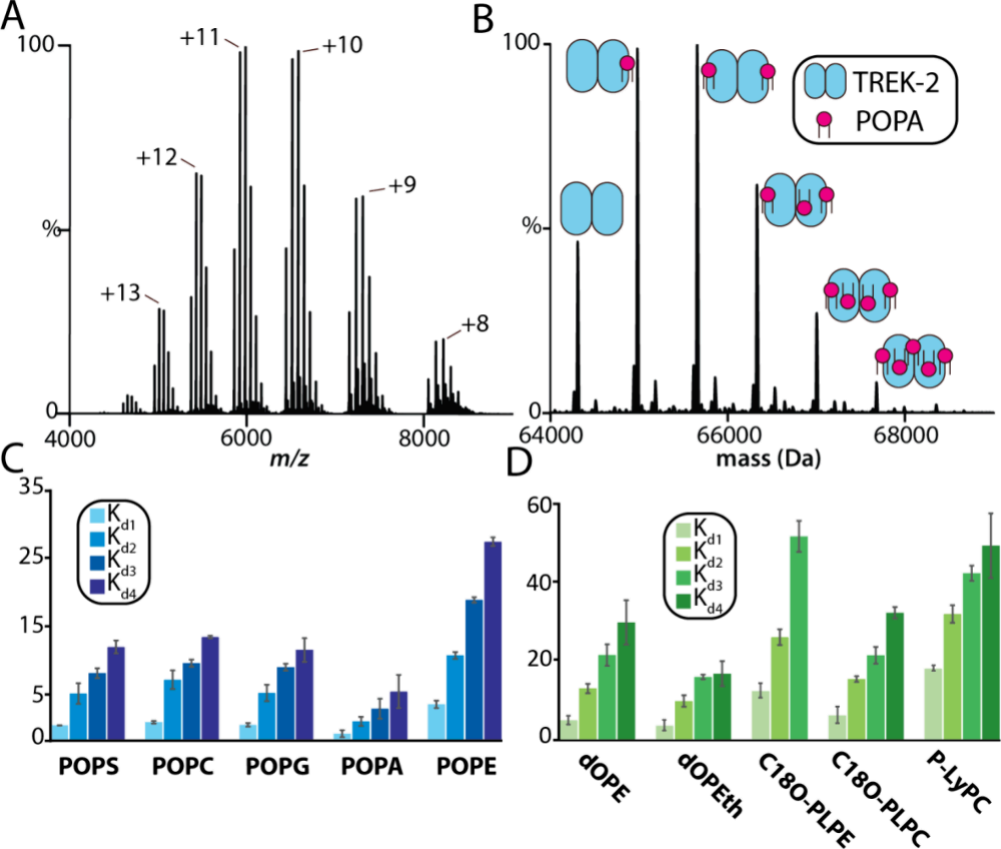
Equilibrium binding constants for lipid binding to TREK2. (A) Native mass spectrum of 0.5 μM TREK2 mixed with 2.5 μM of POPA. (B) Deconvolution of the mass spectrum shown in panel A. (C and D) Equilibrium dissociation constant (*K*_d_) values for lipids containing PO tails and lipids’ alternate chemistries. Lipid abbreviations are provided in Table S1. Reported are the mean and standard deviation (*n* = 3).

Inspired by our previous work on TRAAK–lipid interactions,^9^ we systematically titrated TREK2 with a series of lipids to deduce the channel’s affinity. We began by investigating lipids with PO (1-palmitoyl-2-oleoyl, 16:0–18:1 acyl chains) tails with the following headgroups: PA, phosphatidic acid; PC, phosphatidylcholine; PE, phosphatidylethanolamine; PG, phosphatidylglycerol; and PS, phosphatidylserine. (Figure 2 and Figure S1) For example, the mass spectrum of TREK2 in the C10E5 detergent and in the presence of five equivalents of POPA captures up to five binding events (Figure 2A–B). This and other mass spectra from the titration series are then deconvoluted to determine the mole fraction for apo and lipid bound states. A sequential lipid binding model is then globally fit to the mole fraction data to determine the equilibrium dissociation constants (*K*_d_). The most avidly binding lipid is the anionic lipid POPA with a *K*_d_ for the first binding event (*K*_d1_) of 0.7 ± 0.1 μM. The *K*_d1_ value for POPS (*K*_d1_ = 2.2 ± 0.1 μM) is more than threefold compared to that for POPA and suggests an apparent preference for anionic lipid headgroups. POPC and POPG have slightly higher *K*_d1_ values and are statistically indistinguishable from one another. In the case of POPE, which differs from POPC by three methyl groups, the *K*_d1_ value is significantly higher than that of POPA (*K*_d1_ = 10.4 ± 1.0 μM). Interestingly, although POPE binds with the weakest affinity for TREK2, the affinity is twice that compared to TRAAK.^9^

Since TRAAK has been shown to been sensitive to acyl chain chemistry,^9^ we explored the binding of different PE and PC lipids to TREK2 (Figure 2D). PE containing dO (dioleoyl, 18:1) acyl chains displayed a twofold decrease in the binding constant (*K*_d1_ = 5.2 ± 1.2 μM) compared to its PO counterpart. Phosphatidyl ethanol (PEth) with dO tails, which differs from dOPE by a primary amine, showed no significant changes in the first binding event (*K*_d1_ = 5.5 ± 0.4 μM). Altering the ester linkage at the *sn*-1 position to a vinyl ether, plasmalogens with 18:0, 18:1 acyl chains (C18PLOPE) decreased the binding affinity twofold compared to dOPE (*K*_d1_ = 16.2 ± 2.4 μM). The plasmalogen lipid with a PC headgroup decreased the *K*_d_ value by half (*K*_d1_ = 6.9 ± 0.2 μM). We also investigated lysolipids, such as POPC with the acyl chain at the *sn*-2 tail removed (P-LyPC). P-LyPC had the lowest *K*_d1_ value observed of the lipids studied (*K*_d1_ = 20.7 ± 0.3 μM). These results show that TREK2 is sensitive to lipid chemistry, such as *sn*-1 linkage and acyl chain composition.

**Figure 3:**
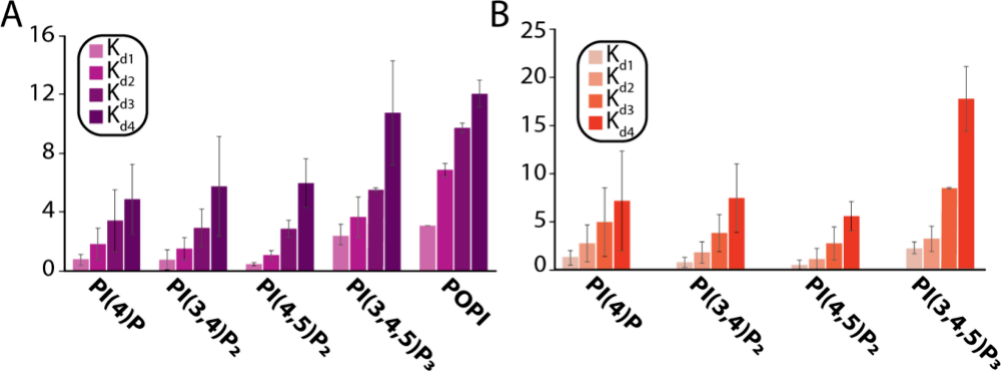
Equilibrium dissociation constants for the interaction of TREK2 with PIPs. (A) Equilibrium dissociation constants (*K*_d_) for PIPs containing dO tails. (B) Equilibrium dissociation constants for PIPs with SA tails. Shown as described in Figure 2.

As signaling lipids have been reported to regulate the TREK subfamily,^14,38^ we investigated phosphatidylinositol (PI) lipids and their phosphorylated forms (Figure 3 and Figure S2). For POPI, the *K*_d1_ value was more than threefold higher than that for POPA (*K*_d1_ = 3.0 ± 0.01 μM). We next examined phosphorylated forms of PI harboring dO (dioleoyl, 18:1) acyl chains. The triphosphorylated lipid, PI(3,4,5)P_3_, bound to TREK2 (*K*_d1_ = 2.4 ± 0.7) and showed a threefold increase compared to that for POPA. The monophosphate lipid, dOPI(4)P, displayed a similar binding affinity (*K*_d1_ = 0.8 ± 0.4 μM) compared to POPA. The addition of a 3′ phosphate to the lipid in dOPI(3,4)P_2_ resulted in a binding affinity that was statistically indistinguishable (*K*_d1_ = 0.8 ± 0.7 μM). In contrast, installation of the 5′ phosphate on dOPI(4)P showed a increase in binding affinity (*K*_d1_ = 0.5 ± 0.1 μM). We also investigated the impact of phosphoinositides containing SA tails (1-stearoyl-2-arachidonoyl, 18:0–20:4 acyl chains). PI(4)P, PI(3,4)P_2_, and PI(3,4,5)P_3_ lipids with SA tails had *K*_d_ comparable to those with dO (Figure 3B). A notable exception was SAPI(3,4,5)P_3_ that displayed a threefold increase in binding affinity (*K*_d1_ = 2.3 ± 0.8 μM) compared to several of the lipids containing dO tails. These results show TREK2 displays a marked preference for the headgroup and acyl chains, which can have a drastic effect on binding affinity.

### Functional Studies of TREK2 in Defined Lipid Environments

**Figure 4:**
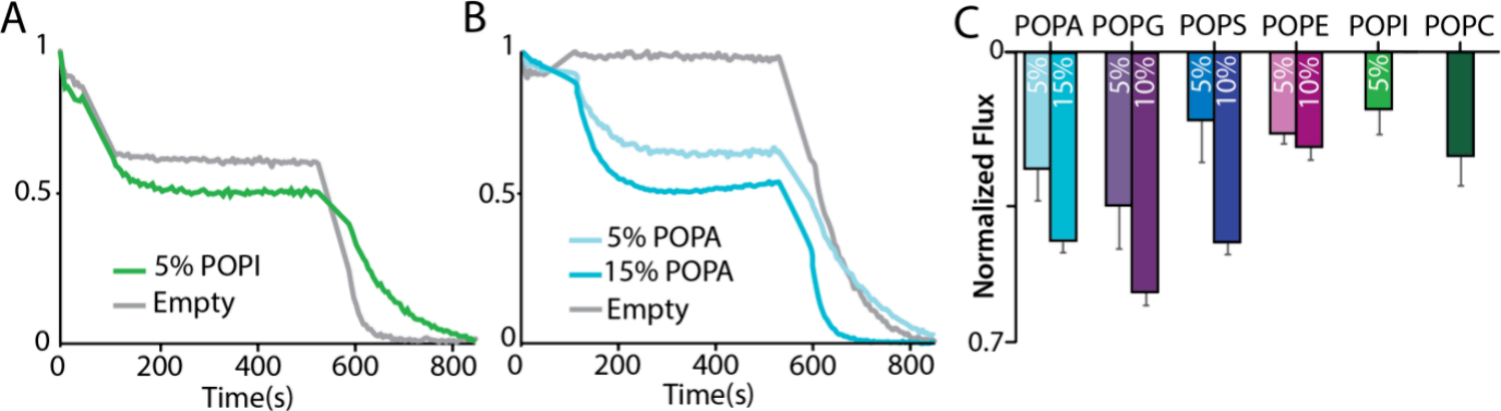
Liposome flux assay of TREK2 in liposomes with defined environments. (A) Flux assay trace with 5% POPI with and without TREK2 incorporated. (B) Flux assay trace with 5% and 15% POPA with and without TREK2 incorporated. (C). Normalized flux assay in POPC with 5–15% of POPA, POPG, POPS, POPE, and POPI.

To better understand the regulation of TREK2 by lipids, we conducted functional assays of the purified channel in defined lipid environments. TREK2 was reconstituted into proteoliposomes consisting of POPC and those doped with different mole fractions of POPA, POPG, POPS, POPE, and POPI (Figure 4). For these experiments, we used the TREK2 samples used in the native MS studies, deglycosylatd protein. POPG and POPA exhibited the largest potassium flux. This is result is interesting as TREK2 exhibited the highest affinity for POPA compared to the other PO lipids. Interestingly, POPI exhibits less flux than POPC liposomes perhaps eluding to an inhibitory effect of POPI on TREK2. When the mole fractions of the doped lipids are increased, the flux values also increase. Notably, an increase from 5% to 10% increases POPS to a flux similar to that of 15% POPA. The increase in PS is surprising since the different *K*_d_ values of POPA are still lower than those of POPS.

## Conclusion

**Figure 5:**
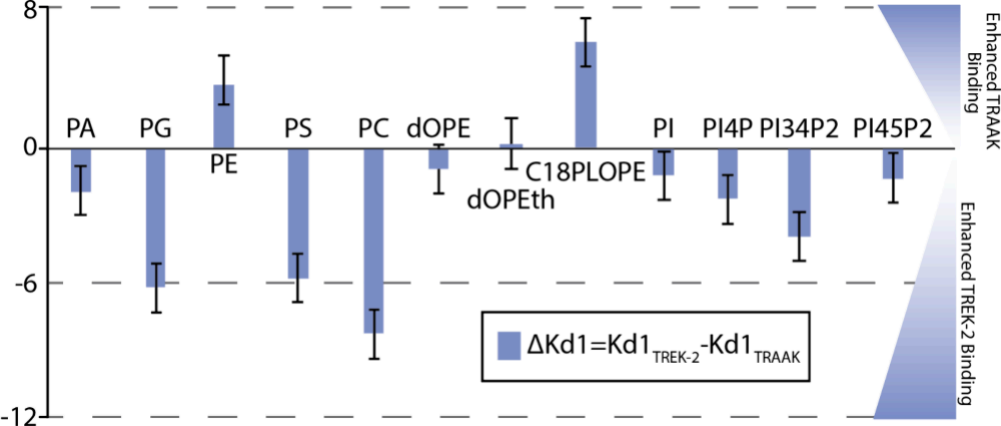
Comparison of the first lipid equilibrium binding constants for TREK2 and TRAAK. The difference between lipid binding to TREK2 minus TRAAK is shown. Values with a positive value indicate increased affinity for TRAAK whereas negative values correspond to better binding to TREK2.

Comparison of the lipid binding affinities for TREK2 and TRAAK sheds light on their preferences for lipids. Visual comparison of the computed electrostatics of the TRAAK (PDB 4WFE) and TREK2 (PDB 4BW5) suggest they have similar properties despite the channels sharing 45% sequence identity (Figure S3). However, comparing the Δ*K*_d1_ values (TRAAK – TREK2), where negative values indicate better binding to TREK2, shows the channels have distinct lipid binding preferences (Figure 5). TREK2 displays a higher binding affinity for the PO lipids, which is surprising due to the high affinity TRAAK has for POPA and activation by this lipid. The C-terminus of TREK2 binds phospholipase D1 (PLD2), which converts PC to PA.^39^ PLD2 would generate a high local concentration of PA to be produced near TREK2, promoting binding of this lipid. There are only two lipids where TRAAK has a higher affinity, a plasmalogen and one of the PIP lipids. The binding affinity of TREK2 for dOPI(4,5)P_2_ was the lowest compared to the other PIPs. In general, TREK2 binds PIPs with higher affinity than TRAAK. TREK2 binds PI(4,5)P_2_ with high affinity, and the binding constant is nearly comparable to what we observed for the lipid binding Kir3.2.^40^ Kir channels have a distinct PIP2 binding site,^41^ and this work provides evidence that TREK2 may also have a distinct binding site for this lipid as well. In short, these findings show TRAAK and TREK2 have distinct lipid binding preferences.

TREK1, a more closely related member of the TREK subfamily, lipid binding affinities have been characterized.^42^ The reported *K*_d_ values for POPG (176.9 μM) and POPA (15.7 μM) are approximately 80 and 17 times higher than the values determined for TREK2, respectively. Both of these lipids were found to be agonists of TREK1, and we find these lipids stimulate TREK2 activity. Potassium efflux was higher for TREK1 compared to TREK2 in liposomes containing POPG. Regarding PI(4,5)P_2_, TREK1 has a slightly higher *K*_d_ value (0.86 μM) compared to that for TREK2 (0.5 μM). It has been shown that TREK1 is markedly activated by PG, especially when compared to PA. Comparing the function of TREK2 and TREK1 in the presence of PA reveals TREK2 is more sensitive to PA and displays higher activity. The function of TREK2 with 5% PA is higher than that of TREK1 with 10% PA.^13^ One of the difficulties of studying membrane protein–lipid interactions like TREK2 is understanding the biological role of these lipids in the function of the channel. While the lipid binding in detergent can determine useful biochemical parameters, such as *K*_d_, this does not necessarily reflect the binding of the lipid in the bilayer but rather a better understanding of how lipid chemistry impacts binding to site(s) on TREK2. However, using native mass spectrometry in combination with functional assays is an excellent way to identify lipids that modulate structure and function. Future directions will incorporate similar studies for membrane proteins reconstituted in liposomes,^43^ where the lipids retained to the ejected membrane protein complex may shed light on affinity in the bilayer.

## Supporting Information Available

Phospholipids and their abbreviations; equilibrium dissociation constants of TREK2–lipid interactions; mass spectra of TREK2 in complex with PO-type lipids and PIPs; comparison of electrostatics of TREK2 and TRAAK (PDF)
