# Supplementary material for: TREK2 Lipid Binding Preferences Revealed by Native Mass Spectrometry
Source: J Am Soc Mass Spectrom. 2024 Jun 6;35(7):1516–22. doi: 10.1021/jasms.4c00112 (PMC11228984; doi:10.1021/jasms.4c00112)
Supplement: Supplementary file 1 — js4c00112_si_001.pdf [file js4c00112_si_001.pdf]

Supporting Information

TREK2 lipid binding preferences revealed by native mass spectrometry

Lauren Stover,[1] Yun Zhu,[1,] Samantha Schrecke,[1] and Arthur Laganowsky[1,*]

[1] Department of Chemistry, Texas A&M University, College Station, TX 77843
*Corresponding Author: ALaganowsky@chem.tamu.edu

Table S1. Phospholipids and their abbreviations.

Abbreviation	IUPAC Name	Mass (Da)
POPA	1-palmitoyl-2-oleoyl-sn-glycero-3-phosphate	673.471
POPC	1-palmitoyl-2-oleoyl-glycero-3-phosphocholine	736.578
POPE	1-palmitoyl-2-oleoyl-sn-glycero-3-phosphoethanolamine	717.531
POPG	1-palmitoyl-2-oleoyl-sn-glycero-3-phospho-(1'-rac-glycerol)	747.507
POPS	1-palmitoyl-2-oleoyl-sn-glycero-3-phospho-L-serine	760.503
POPI	1-palmitoyl-2-oleoyl-sn-glycero-3-phosphoinositol	854.100
dOPE	1,2-dioleoyl-sn-glycero-3-phosphoethanolamine	743.547
dOPEth	1,2-dioleoyl-sn-glycero-3-phosphoethanol	727.518
C18O-PLPE	1-(1Z-octadecenyl)-2-oleoyl-sn-glycero-3-phosphoethanolamine	729.567
C18O-PLPC	1-(1Z-octadecenyl)-2-oleoyl-sn-glycero-3-phosphocholine	771.614
P-LyPC	1-palmitoyl-2-hydroxy-sn-glycero-3-phosphocholine	495.332
dOPI(4)P	1,2-dioleoyl-sn-glycero-3-phospho-(1'-myo-inositol-4'-phosphate)	940.577
dOPI(3,4)P_2	1,2-dioleoyl-sn-glycero-3-phospho-(1'-myo-inositol-3',4'-bisphosphate)	1019.569
dOPI(4,5)P_2	1,2-dioleoyl-sn-glycero-3-phospho-(1'-myo-inositol-4',5'-bisphosphate)	1019.569
dOPI(3,4,5)P_3	1,2-dioleoyl-sn-glycero-3-phospho-(1'-myo-inositol-3',4',5'-trisphosphate)	1098.562
SAPI(4)P	1-stearoyl-2-arachidonoyl-sn-glycero-3-phospho-(1'-myo-inositol-4'-phosphate)	964.577

SAPI(3,4)P_2	1-stearoyl-2-arachidonoyl-sn-glycero-3-phospho-(1'-myo-inositol-3',4'-bisphosphate)	1043.568
SAPI(4,5)P_2	1-stearoyl-2-arachidonoyl-sn-glycero-3-phospho-(1'-myo-inositol-4',5'- bisphosphate)	1043.568
SAPI(3,4,5)P_3	1-stearoyl-2-arachidonoyl-sn-glycero-3-phospho-(1'-myo-inositol-3',4',5'-trisphosphate)	1122.562

Table S2. Equilibrium dissociation constants of TREK2-lipid interactions.

	KD_1 (μM)	KD_2 (μM)	KD_3 (μM)	KD_4 (μM)	R^2	X^2
POPA	0.7±0.1	2.1±0.3	3.6±1.0	5.5±1.7	0.99	0.05
POPC	2.8±0.3	7.2±0.5	11.0±0.5	16.8±1.3	0.96	0.14
POPE	10.4±1.0	21.7±0.8	30.8±0.5	40.2±7.8	0.98	0.07
POPG	2.6±0.5	6.5±0.6	10.5±1.9	14.8±3.3	0.98	0.09
POPS	2.2±0.1	5.9±0.7	9.0±1.1	11.46±1.2	0.98	0.08
POPI	3.0±0.01	6.8±0.4	9.6±0.3	11.9±0.9	0.99	0.03
dOPE	5.2±1.2	9.6±1.0	14.2±0.9	19.9±2.3	0.99	0.04
dOPEth	5.5±0.4	10.0±0.2	13.8±0.9	17.3±4.2	0.99	0.08
C18O-PLPE	16.2±2.4	31.1±3.5	57.6±7.2	-	0.97	0.12
C18O-PLPC	6.9±0.2	14.6±1.4	21.6±1.2	29.9±1.5	1.0	0.01
P-LyPC	20.7±0.3	34.4±1.4	42.1±4.1	44.5±6.8	0.99	0.02
dOPI(4)P	0.8±0.4	1.8±1.1	3.4±2.1	4.8±2.3	0.98	0.03
dOPI(3,4)P_2	0.8±0.7	1.5±0.8	2.9±1.3	5.7±3.4	0.98	0.04
dOPI(4,5)P_2	0.5±0.1	1.1±0.3	2.9±0.6	5.9±1.6	0.98	0.04
dOPI(3,4,5)P_3	2.4±0.7	3.7±1.3	5.4±0.1	10.6±3.4	0.96	0.08
SAPI(4)P	1.2±0.8	2.7±2.0	4.9±3.5	7.0±5.3	0.97	0.11
SAPI(3,4)P_2	0.7±0.5	1.8±1.0	3.6±2.0	7.4±3.5	0.96	0.07
SAPI(4,5)P_2	0.4±0.5	1.1±1.1	2.7±1.8	5.4±1.7	0.96	0.12
SAPI(3,4,5)P_3	2.3±0.8	4.6±1.7	9.3±3.9	17.0±10.3	0.98	0.05

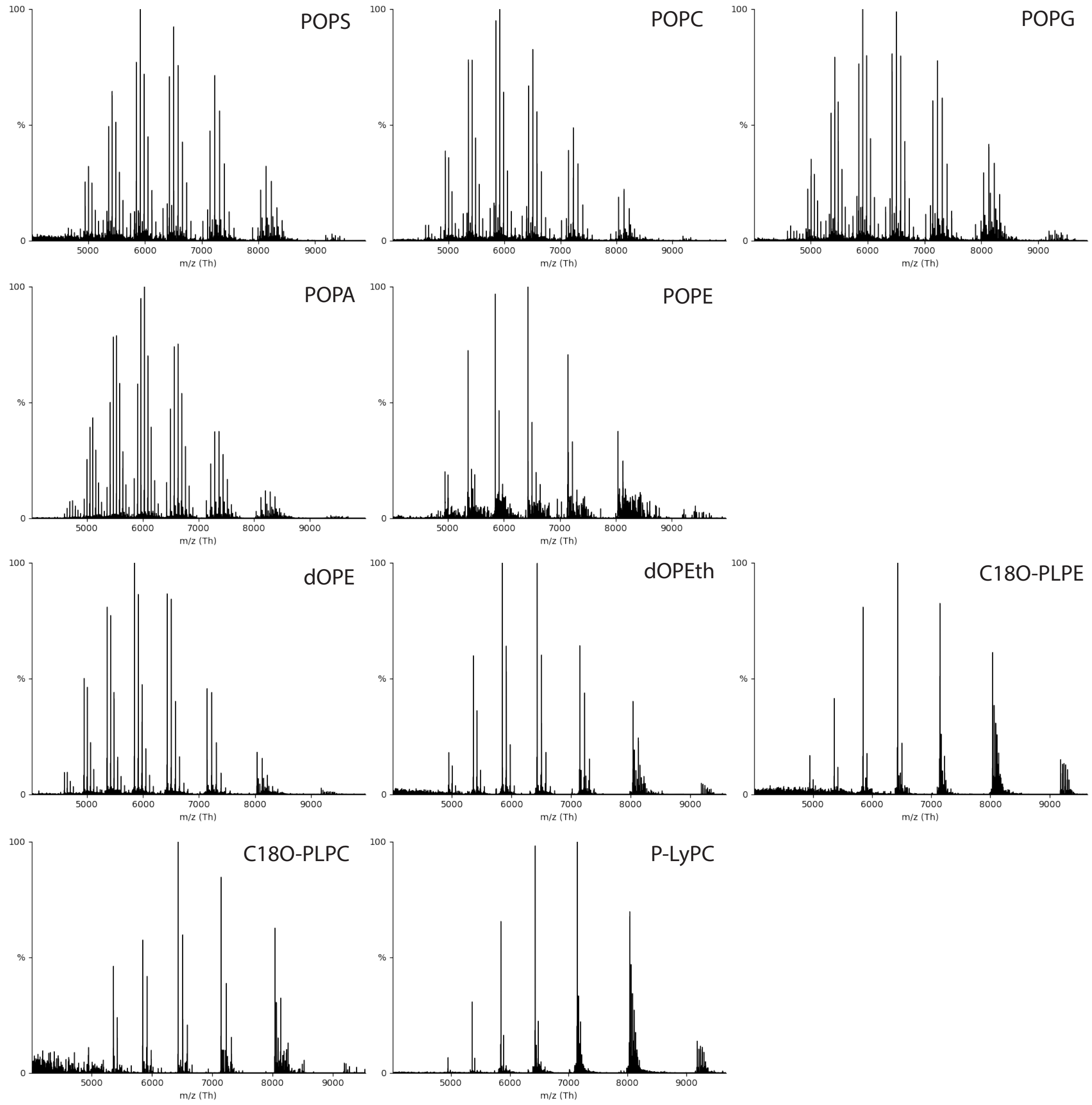

Figure S1. Mass spectra of TREK2 in complex with PO-type lipids. Representative mass spectra correspond to the addition of 5 µM of the given lipid.

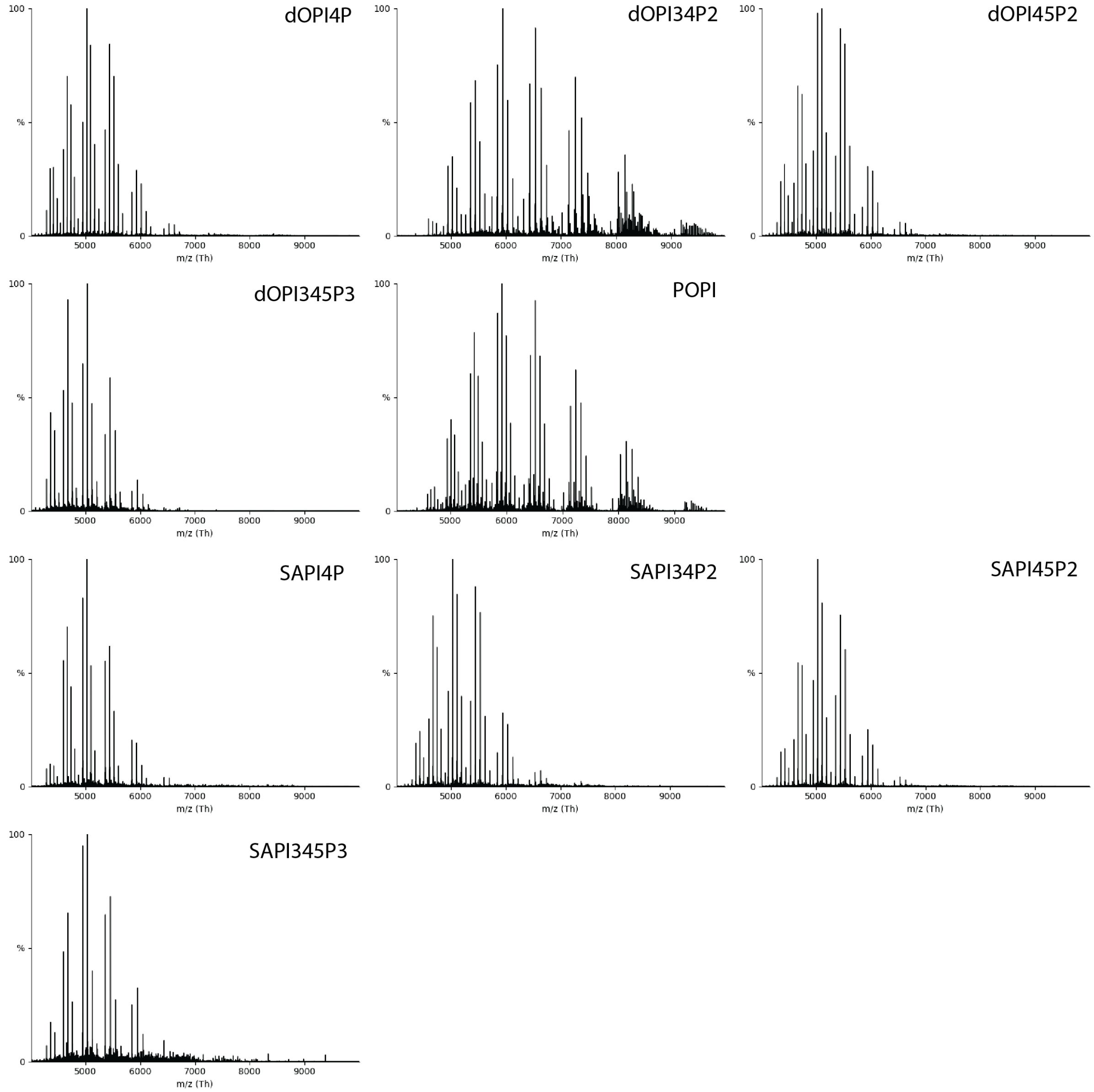

Figure S2. Mass spectra of TREK2 in complex with PIPs. Representative mass spectra correspond to the addition of 5 μM of the given lipid.

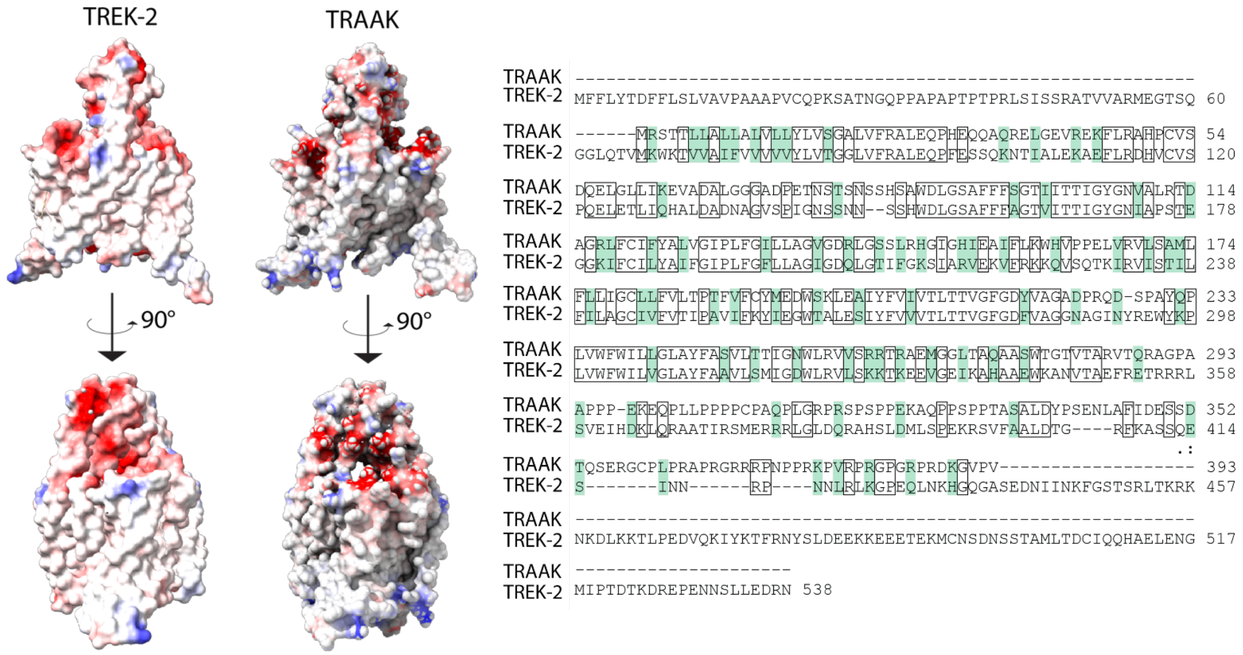

Figure S3. Comparison of Electrostatics of TREK2 and TRAAK. Shown in the left panel is the electrostatic potential comparison between TREK-2 and TRAAK. Positive and negative are colored blue and red, respectively. Figure computed using ChimeraX.[1] Shown to the right is the sequence comparison between TREK-2 and TRAAK, the boxes indicate identical residues while the green highlight indicates similar residue chemistry.

Supporting References

1 Pettersen, E. F. *et al.* UCSF ChimeraX: Structure visualization for researchers, educators, and developers. *Protein Sci* **30**, 70-82 (2021). https://doi.org:10.1002/pro.3943
